# Supplementary material for: Flow-induced elongation of von Willebrand factor precedes tension-dependent activation
Source: Nat Commun. 2017 Aug 23;8:324. doi: 10.1038/s41467-017-00230-2 (PMC5567343; doi:10.1038/s41467-017-00230-2)
Supplement: Supplementary file 1 — Supplementary Information [file 41467_2017_230_MOESM1_ESM.pdf]

File name: Supplementary Information
Description: Supplementary Figures

File name: Supplementary Movie 1
Description: VWF elongation. VWF concatemers in cycles of start- and stop-flow with reversal of direction at the indicated wall shear stresses (10 ms exposure every second). The field of view includes the molecule shown in Fig. 1c. Scale bar, 5 µm.

File name: Supplementary Movie 2
Description: VWF relaxation kinetics. VWF concatemers were subjected to cycles of 1280 dyn·cm-2 wall shear stress and flow cessation in forward and backward flow directions in buffer with 60% w/w sucrose and 150 mM NaCl. The field of view includes the molecule shown in Fig. 2a
Images were taken at 20 fps. Scale bar, 5 µm.

File name: Supplementary Movie 3
Description: GPIbα binding to VWF in flow. Tethered VWF concatemers (Alexa-488 channel in green pseudocolor) were subjected to on- and off-flow cycles at indicated wall shear stresses in the presence of free GPIbα (Alexa-647 channel in magenta pseudocolor). The example is with 20 nM GPIbα in 10 mM NaCl (Supplementary Fig. 3b). Movie was recorded with 10 ms exposure every 300 ms. Scale bar, 5 µm.

File name: Peer Review File
Description:

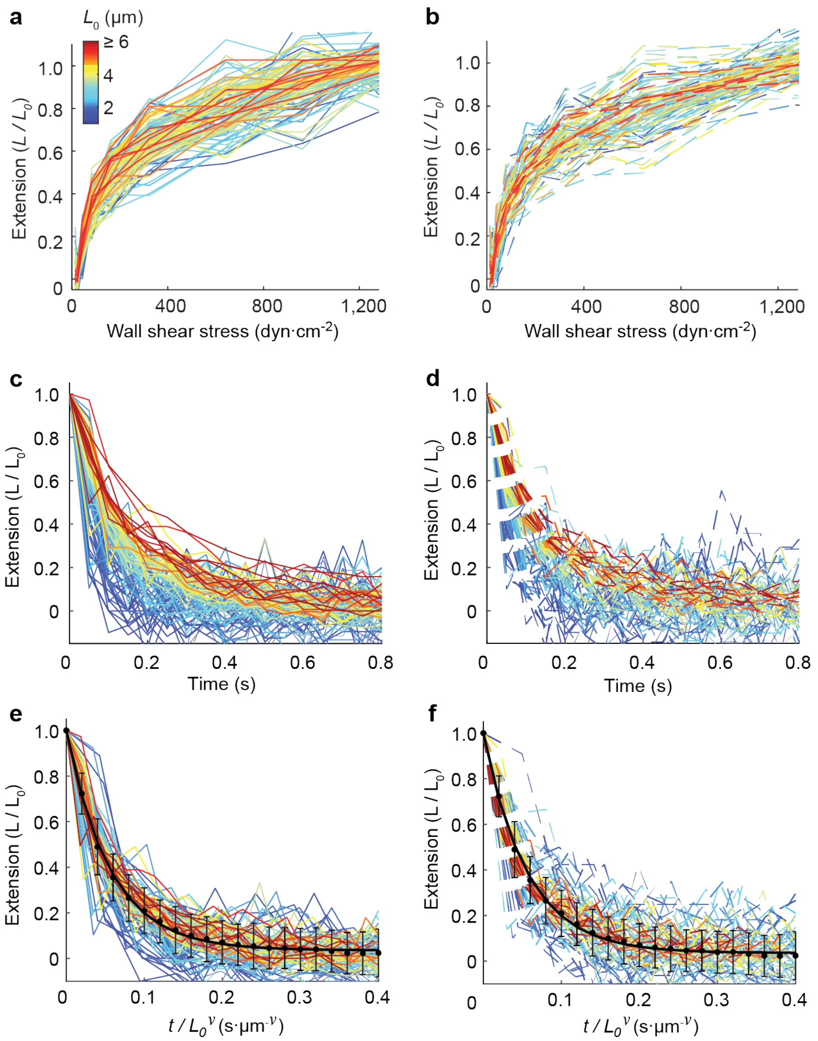

Supplementary Figure 1. Mechanical response of VWF under forward and backward shear flows. Data are normalized to length at 1280 dyn·cm^{-2} (L_0, color key). **(a-b)**. VWF concatemer extension (N = 112 concatemers) vs. wall shear stress under forward **(a)** and backward **(b)** flow in Fig. 1d. **(c-f)**. VWF dynamics (N = 94 concatemers) during relaxation from forward **(c)** and backward **(d)** flows in Fig. 2b. Fits to the equations in Fig. 2d shown (black solid lines) for VWF relaxation from forward **(e)** or backward **(f)** flows for all the 94 VWF concatemers in Fig. 2d. Points and error bars show mean and sd for all concatemers relaxed from flow in both directions.

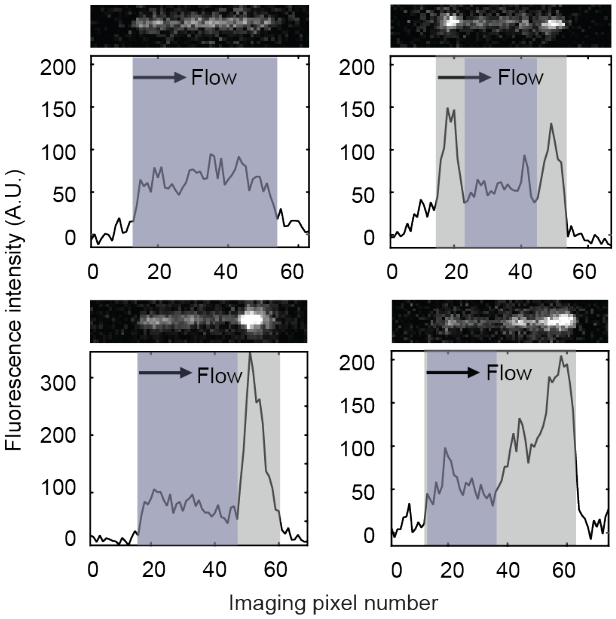

Supplementary Figure 2. Representative images and fluorescence intensity traces of VWF. Examples of VWF-Alexa Fluor 488 fluorescence intensity profiles (black traces) for extended VWF molecules in buffer with 150 mM NaCl. Entire (grey plus purple) and low (purple) intensity regions exemplify the regions selected for calculating the average extension per VWF monomer in Fig. 1f. The fluorescence image of each VWF molecule is shown above its intensity profile.

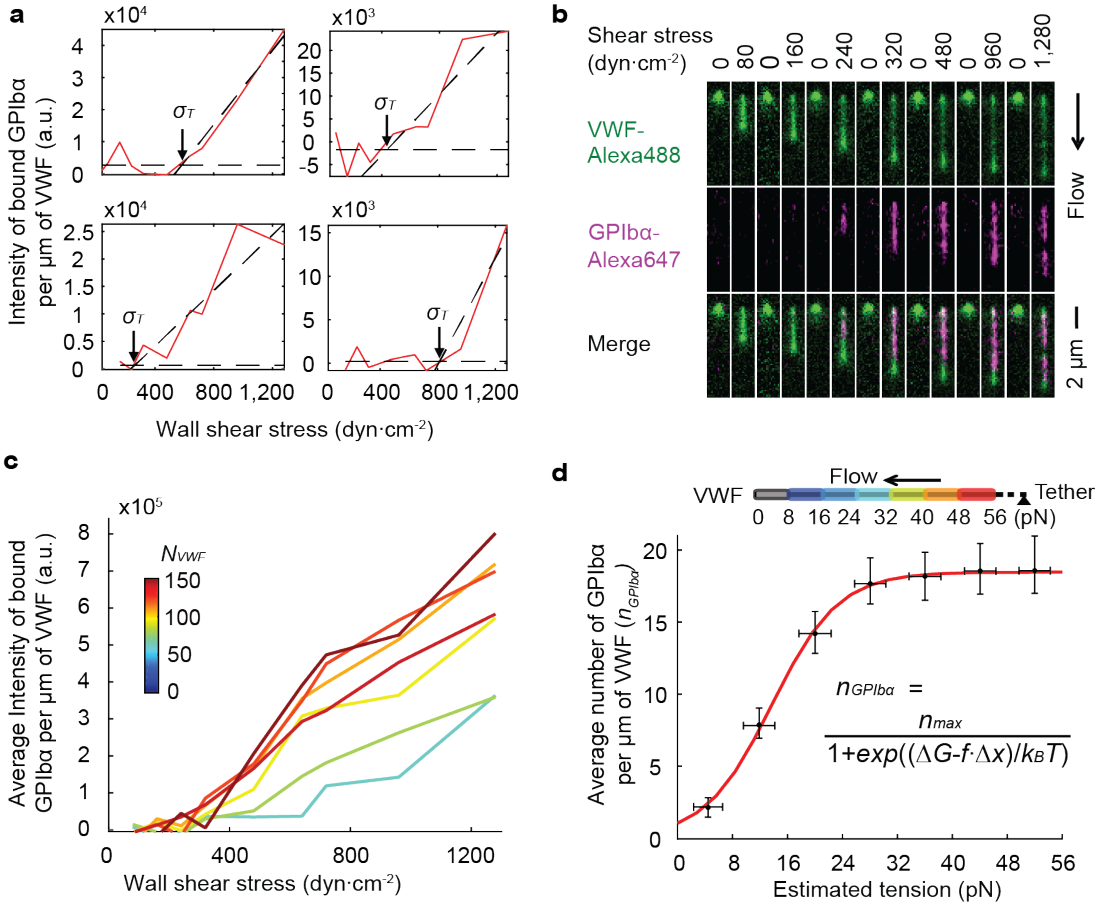

Supplementary Figure 3. VWF-GPIbα binding in presence of 20 nM free GPIbα in 10 mM NaCl. (a) Examples of determining the critical shear stress σ_T by the intercept between the linear fit (diagonal dashed line) of the increasing region of bound GPIbα intensity curve (red line) and the background intensity (horizontal dashed line). **(b)** An example of Alexa Fluor 488-labeled VWF molecule extension and reversible binding to 20 nM Alexa Fluor 647-labeled GPIbα during cycling between stasis and a range of wall shear stresses. **(c)** Average fluorescence intensity of bound GPIbα per μm of VWF under increasing wall shear stress. Every 30 VWF concatemers from 210 molecules are grouped into size bins in order of ascending N_{VWF}. Colored lines (see color key) show the average bound GPIbα intensity in each size bin. **(d)** Equilibrium binding of GPIbα per μm ($n_{GPIb\alpha}$) in tension bins along the length of VWF concatemers extended at 1280 dyn·cm^{-2}. Data are mean for 100 concatemers that were long enough to include all 7 tension bins. Error bars are sd for tension and 95% confidence intervals for the average number of GPIbα. Red line: two-state model fit (inset formula).

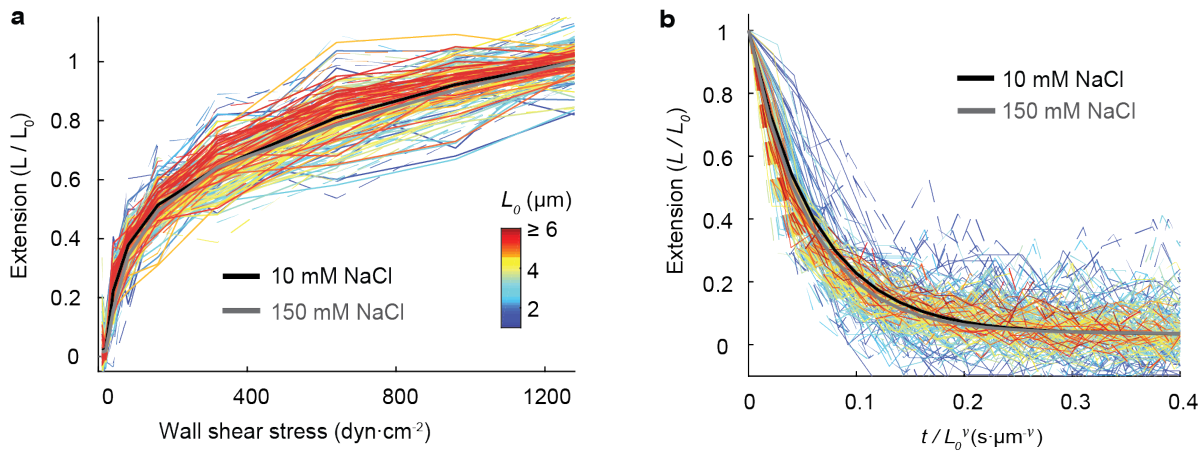

Supplementary Figure 4. Mechanical response of VWF under shear flow in 10 mM NaCl. Data are normalized to length at 1280 dyn·cm^{-2} (L_0, color key). **(a)** VWF concatemer extension (N = 91 concatemers) vs. wall shear stress under forward (solid lines) and backward (dashed lines) flow. Black line is average extension in 10 mM NaCl and grey line is average extension in 150 mM NaCl (Fig. 1d). **(b)** VWF relaxation dynamics in 10 mM NaCl with 60 % (w/w) sucrose. Black (10 mM NaCl) and grey (150 mM NaCl) lines show fits to the equations in Fig. 2d. Fitting parameters with 95% confidence intervals for 10 mM NaCl are a=0.97±0.01, δ=0.062±0.006 s·μm$^{-\nu}$, ν=0.42±0.09.

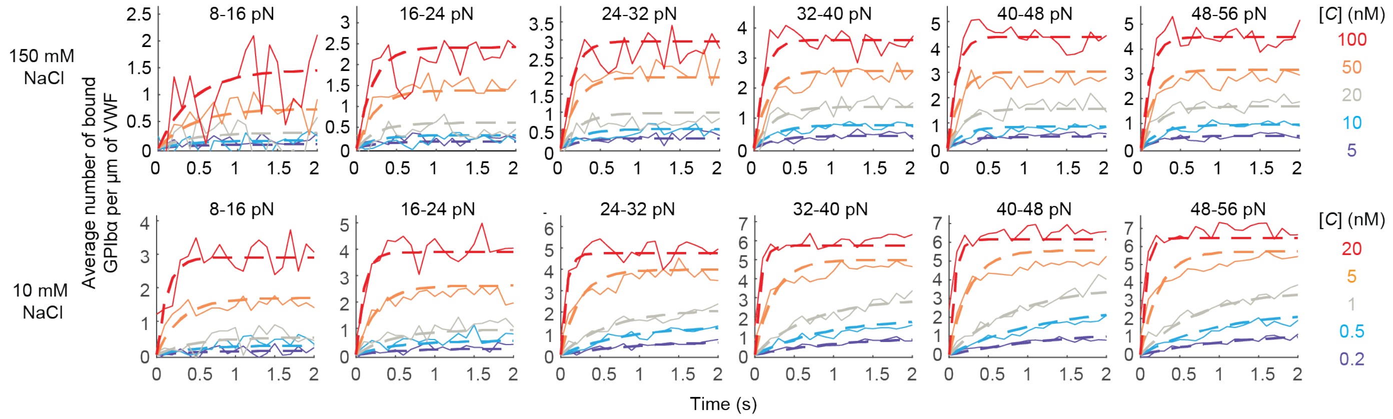

Supplementary Figure 5. VWF-GPIbα binding kinetics under flow. The data correspond to those analyzed in Fig. 3f-g. GPIbα binding kinetics (solid lines) were measured at 10 fps after shift from 0 to 1280 dyn·cm^{-2} at the different GPIbα concentrations [*C*] (color coded to the right) in six VWF tensile force bins. Fits to equation (6) (dashed lines) are shown in corresponding colors. Numbers of VWF concatemers measured at each concentration of GPIbα were for 150 mM NaCl: 129 (5 nM), 108 (10 nM), 92 (20 nM), 141 (50 nM), and 117 (100 nM), and were for 10 mM NaCl: 93 (0.2 nM), 87 (0.5 nM), 102 (1 nM), 141 (5 nM), and 78 (20 nM).
